# Supplementary material for: Transmissibility of COVID-19 depends on the viral load around onset in adult and symptomatic patients
Source: PLoS One. 2020 Dec 9;15(12):e0243597. doi: 10.1371/journal.pone.0243597 (PMC7725311; doi:10.1371/journal.pone.0243597)
Supplement: S1 Appendix — (DOCX) [file pone.0243597.s001.docx]

The telephone interview questionnaire

| Date |  | Interviewer |  |
| --- | --- | --- | --- |

| Categories | | | | Details |
| --- | --- | --- | --- | --- |
| Patient ID | | | |  |
| Demographics | | | | |
|  | Age | | | years old |
|  | Sex | | |  |
|  | Underlying diseases | | |  |
| Clinical presentation | | | | |
| For the patients admitted to other hospitals only, | | | |  |
|  | Symptoms | | |  |
|  | The date of symptom onset | | |  |
|  | The date of the initial sample collection | | |  |
|  | Hospitalization | | |  |
|  | Name of hospital | | |  |
|  | The date of admission | | |  |
|  | The date of the first negative RT-qPCR test result | | |  |
|  | The date of discharge | | |  |
|  | Given supplemental oxygen | | |  |
|  | Admission to intensive care unit | | |  |
|  | Ventilator | | |  |
| Contact tracing | | | | |
|  | Contact with a person with COVID-19 (probable or confirmed) within the last 14 days | | |  |
|  | The infection route considered by the patients themself | | |  |
|  | For asymptomatic cases only, specify the reason for the COVID-19 test | | |  |
| Details of close contacts | | | | |
|  | The number of household contact | | |  |
|  | Of these, the number of household contacts tested for COVID-19 | | |  |
|  | Of these, the number of confirmed COVID-19 patients | | |  |
|  |  | Name/Relationship | The date of symptom onset | The date of the initial sample collection |
|  |  |  |  |  |
|  |  |  |  |  |
|  |  |  |  |  |
|  | The number of work-related close contacts in the workplace | | |  |
|  | Of these, the number of work-related close contacts tested for COVID-19 | | |  |
|  | Of these, the number of confirmed COVID-19 patients | | |  |
|  |  | Name/Relationship | The date of symptom onset | The date of the initial sample collection |
|  |  |  |  |  |
|  |  |  |  |  |
|  |  |  |  |  |
|  | The number of other close contacts | | |  |
|  | Of these, the number of other close contacts tested for COVID-19 | | |  |
|  | Of these, the number of confirmed COVID-19 patients | | |  |
|  |  | Name/Relationship | The date of symptom onset | The date of the initial sample collection |
|  |  |  |  |  |
|  |  |  |  |  |
|  |  |  |  |  |
