# Supplementary material for: Transmissibility of COVID-19 depends on the viral load around onset in adult and symptomatic patients
Source: PLoS One. 2020 Dec 9;15(12):e0243597. doi: 10.1371/journal.pone.0243597 (PMC7725311; doi:10.1371/journal.pone.0243597)
Supplement: S2 Appendix — (DOCX) [file pone.0243597.s002.docx]

調査票

| 調査日 |  | 調査者 |  |
| --- | --- | --- | --- |

| 項目 | | | | 詳細 | |
| --- | --- | --- | --- | --- | --- |
| 患者ID | | | |  | |
| 患者背景 | | | | | |
|  | 年齢 | | | 歳 | |
|  | 性別 | | |  | |
|  | 基礎疾患 | | |  | |
| 臨床経過 | | | | | |
| 当院以外に入院した患者のみ対象 | | | |  | |
|  | 症状 | | |  | |
|  | 発症日 | | |  | |
|  | PCR検査初回陽性検体採取日 | | |  | |
|  | 入院の有無 | | |  | |
|  | 入院した医療機関名 | | |  | |
|  | 入院日 | | |  | |
|  | PCR検査が初めて陰性化した検体採取日 | | |  | |
|  | 退院日 | | |  | |
|  | 酸素投与の有無 | | |  | |
|  | ICU入室の有無 | | |  | |
|  | 人工呼吸器使用の有無 | | |  | |
| 接触者調査 | | | | | |
|  | 発症14日以内のCOVID-19疑いもしくは確定患者との接触歴の有無 | | |  | |
|  | 患者自ら予想される感染経路 | | |  | |
|  | 無症状の場合、PCR検査を受けた理由 | | |  | |
| 濃厚接触者 | | | | | |
|  | 同居家族の数 | | |  | |
|  | 同居家族のうち、濃厚接触者としてPCR検査を受けた人数 | | |  | |
|  | そのうち陽性であった人数（いれば下記に詳細を記入） | | |  | |
|  |  | 名前及び関係性 | 発症日 | | 初回陽性検体採取日 |
|  |  |  |  | |  |
|  |  |  |  | |  |
|  |  |  |  | |  |
|  | 職業での濃厚接触者数 | | |  | |
|  | 職場のうち、濃厚接触者としてPCR検査を受けた人数 | | |  | |
|  | そのうち陽性であった人数（いれば下記に詳細を記入） | | |  | |
|  |  | 名前及び関係性 | 発症日 | | 初回陽性検体採取日 |
|  |  |  |  | |  |
|  |  |  |  | |  |
|  |  |  |  | |  |
|  | その他の濃厚接触者の数 | | |  | |
|  | そのうち陽性であった人数（いれば下記に詳細を記入） | | |  | |
|  |  | 名前及び関係性 | 発症日 | | 初回陽性検体採取日 |
|  |  |  |  | |  |
|  |  |  |  | |  |
|  |  |  |  | |  |
